# Characterizing social motivation deficits in treatment‐resistant schizophrenia: Evidence from social incentive performance

**DOI:** 10.1111/pcn.70094

**Published:** 2026-06-17

**Authors:** Simon S.Y. Lui, Jasmine W.S. Chan, Jason L.F. Chan, Kimi H.Y. Lam, Lok‐Yin Choi, Raisie W.K. Wong, Perry B.M. Leung, Na Zhan, Jason W.Y. Wong, Mindi W.Y. Chiu, Jenny P.H. Lam, Raymond C.K. Chan

**Affiliations:** ^1^ Department of Psychiatry, School of Clinical Medicine The University of Hong Kong Hong Kong China; ^2^ Department of Psychiatry Queen Mary Hospital Hong Kong China; ^3^ Castle Peak Hospital Hong Kong China; ^4^ Neuropsychology and Applied Cognitive Neuroscience Laboratory, Department of Psychiatry, School of Clinical Medicine The University of Hong Kong Hong Kong China

**Keywords:** negative symptoms, second‐generation scales, social motivation, treatment‐resistant schizophrenia

## Abstract

**Aim:**

The Treatment‐Response‐and‐Resistance‐in‐Psychosis (TRRIP) criteria recognizes negative symptoms as defining features for treatment‐resistant schizophrenia (TRS). Social motivation refers to the desire to pursue social reward (and to avoid social punishment). Impaired social motivation is related to negative symptoms but has seldom been studied in TRS patients. We aimed to characterize social motivation processing in TRS patients and examine the impacts of negative symptoms on social motivation processing.

**Methods:**

We recruited 60 TRS patients, 60 remitted schizophrenia patients and 60 controls. In the Social Incentive Delay (SID) task, participants had to hit the targets within the response window to seek positive/neutral/negative social outcomes in emoji formats; and they also rated their emotions during the anticipation and receipt of social outcomes. Generalized‐estimation‐equation (GEE) models examined the impacts of TRS/remitted schizophrenia, negative symptoms, and the interaction between clinical‐group‐status and cue‐valence on the SID performance, that is, hit‐or‐not, anticipatory pleasure, and consummatory pleasure.

**Results:**

The interaction between TRS status and social reward/punishment significantly predicted anticipatory pleasure, implicating that TRS patients' anticipatory pleasure was less subject to social reward/punishment compared to controls. Moreover, the interaction between TRS and social reward significantly predicted consummatory pleasure, implicating that TRS patients' consummatory pleasure was less subject to social reward compared to controls. Negative symptoms significantly predicted the SID accuracy in clinical participants, supporting its relationship with social motivation processing.

**Conclusion:**

TRS patients have altered social motivation processing, affecting anticipatory and consummatory pleasure. Social motivation deficit may be an intervention target for negative symptoms and social functioning impairments in TRS patients.

Treatment‐resistant schizophrenia (TRS) is characterized by failure to respond to at least two non‐clozapine antipsychotic medications, persistent psychotic symptoms, and functional deterioration.^1^, ^2^ The important role of negative symptoms in TRS patients has gained growing attention. On the one hand, the Treatment Response and Resistance in Psychosis (TRRIP) criteria have now recognized negative symptoms as one of the defining features for treatment‐resistance.^1^ On the other hand, many TRS patients respond partially to clozapine and suffer from negative symptoms, severely affecting their social functioning.^3^ The ‘motivation and pleasure’ (MAP) and ‘expressivity’ (EXP) domains of negative symptoms both cover social and non‐social aspects.^3^, ^4^, ^5^ A recent study measured negative symptoms in TRS patients using the Clinical Assessment Interview for Negative Symptoms (CAINS),^6^ and reported that TRS patients having higher levels of CAINS‐MAP and EXP symptoms were more impaired in social functioning than their TRS counterparts with lower levels of these symptoms.^7^ Moreover, another recent study^8^ measured the subdomains of ‘diminished expressivity’ and ‘social anhedonia’ of negative symptoms in TRS patients using the Positive and Negative Syndrome Scales (PANSS),^9^ and reported that only the ‘social anhedonia’ subdomain predicted patients' social functioning. However, symptom rating scales such as the CAINS and the PANSS cannot tap into the cognitive mechanisms for social anhedonia in TRS patients.

In psychology, social motivation refers to the desire to pursue and maintain social relationships^10^ (which is one of the core psychological needs of humankind),^11^ and covers both ‘approach’ motivation (i.e. social drive for seeking positive social reward such as praise) and ‘avoidance’ motivation (i.e. social drive for avoid negative social reward such as blame/rejection).^12^ As such, social motivation is considered an extrinsic, autonomous motivation according to the self‐determination theory.^11^ The construct of social motivation differs from social cognition, which is defined as the ability to process social‐salient information to understand and interact with the social world.^13^ Empirical research using experimental/behavioral paradigms suggests that schizophrenia patients have impaired social motivation.^14^, ^15^ For instance, schizophrenia patients anticipate less pleasure from positive social outcomes, and expend less effort to pursue positive social outcomes (or to avoid negative social outcomes) than healthy people.^14^ Moreover, a recent study administered an effort‐based decision‐making paradigm to schizophrenia patients, and found that they were less willing to choose high‐demand but high‐reward social scenarios than healthy people, suggesting a diminished motivation to positive social reward.^15^ To our knowledge, no study to‐date has been conducted to tap into social reward processing and motivation in the TRS population; and it remains unclear whether social motivation processing in TRS patients would differ from remitted schizophrenia patients. Moreover, further research is warranted to investigate whether negative symptoms in TRS patients would affect their social motivation processing. These unresolved issues constitute barriers to development of interventions targeting at social motivation for TRS patients.^3^

To address these knowledge gaps, we conducted this comparative study. We aimed to examine social motivation processing in TRS patients, relative to remitted schizophrenia and healthy people. We also aimed to examine the impacts of negative symptoms on social motivation processing in TRS patients. Given that TRS patients usually have difficulty to carry out complicated motivational tasks (such as the effort‐based decision‐making task using social scenarios),^15^ we chose a relatively simple experimental paradigm, that is, the Social Incentive Delay (SID) task.^16^, ^17^ The SID task taps into patients' motivational processing to approach positive social outcomes (i.e. social *approach* motivation) and to avoid negative social outcomes (i.e. social *avoidance* motivation). The SID task is a variant of the classic Monetary Incentive Delay (MID) task,^17^, ^18^ which has been extensively used in schizophrenia samples to tap into motivation processing.^19^, ^20^, ^21^ This study made three hypotheses. First, we hypothesized that TRS patients would differ in social motivation processing from remitted schizophrenia patients and healthy controls. Second, we hypothesized that negative symptoms would have impacts on social motivation processing in both TRS and remitted schizophrenia patients. Third, we hypothesized that TRS patients would have altered social motivation processing relative to remitted schizophrenia patients and healthy controls, even after controlling for the effects of negative and other symptoms.

## Methods

### Participants

We recruited 60 TRS patients and 60 remitted schizophrenia patients from the early psychosis intervention clinics and step‐down clinics at Castle Peak Hospital (CPH), Hong Kong. Our schizophrenia sample had shorter illness duration than the chronic samples (>10 years of illness) recruited in prior research on negative symptoms in TRS patients.^7^, ^8^ The inclusion criteria for clinical patients were: (i) DSM‐IV schizophrenia^22^; (ii) aged 16–55; (iii) ethnic Chinese; and (iv) able to speak and read Chinese. Moreover, TRS participants must have received clozapine, or have non‐response to at least two different non‐clozapine antipsychotics despite adequate duration (>6 weeks) and dosage (>600 mg/day chlorpromazine equivalence).^1^ Nevertheless, we did not stipulate that our participants with TRS should have at least 12‐weeks persistent psychotic symptoms, >80% medication adherence, and at least moderate levels of functional impairments according to the TRRIP criteria.^1^ For remitted schizophrenia participants, we followed the symptom severity domain of the Andreasen's remission criteria,^23^ that is, (i) having scored ≤3 in the P1, P2, P3, N1, N4, N6, G5 and G9 items of the Positive and Negative Syndrome Scale (PANSS).^9^ Given our cross‐sectional design, we did not stipulate the duration domain (6 consecutive months) of the Andreasen's remission criteria in our clinical comparison group.^23^ The exclusion criteria included: (i) an IQ of <70; (ii) a history of head injury resulting in >30 min of loss of consciousness; (iii) neurological disorders; (iv) substance abuse in the past 12 months; (v) electroconvulsive therapy in the past 6 months; and (vi) uncorrected hearing or visual impairments. Moreover, we recruited healthy controls from nearby community, using the same exclusion criteria applicable to clinical participants. Healthy controls received a structured interview by qualified psychiatrists to ensure the absence of personal and family history of schizophrenia spectrum disorder.

This study was approved by the Ethics Committee of New Territories West Cluster (Approval number: PAED‐2023‐044), and carried out in accordance with the World Medical Association Declaration of Helsinki. All participants provided written informed consent. After the assessments, participants were given a supermarket coupon valued HK$50 (approximately US$6) as subjective incentive, regardless of SID performance.

### Clinical assessments

Clinical participants' diagnosis of schizophrenia was ascertained using the Structured Interview for DSM‐IV (SCID‐I).^24^ We gathered their demographics and clinical variables (e.g. date of service entry, psychosis onset, number of previous psychiatric admissions, and the current type and dose of psychotropic medications) in the structured interviews, supplemented by reviews of medical records. The antipsychotic medication dosage was converted to daily defined dose (DDD) of olanzapine equivalence.^25^ We also estimated the anticholinergic medication burden,^26^ based on the current antipsychotic medications at the time of assessments. Participants' IQ was estimated using the Test of Nonverbal Intelligence‐ Fourth Edition (TONI‐4).^27^

Besides the PANSS,^9^ we administered the CAINS^6^, ^28^, ^29^ to evaluate clinical symptoms. The CAINS is a 13‐item second‐generation scale, consisting of the motivation and pleasure subscale (MAP) and the expressivity subscale (EXP). In addition, we measured parkinsonism features using the Extrapyramidal Side‐effect Rating Scale (ESRS)^30^ and the Abnormal Involuntary Movement Scale (AIMS).^31^ We also measured depressive symptoms using the 17‐item Hamilton Rating Scale for Depression (HAM‐D).^32^ The Drug Attitude Inventory (DAI)^33^ and the Medication Adherence Rating Scale (MARS)^34^ were used to measure medication adherence. Lastly, the Social and Occupational Functioning Assessment Scale (SOFAS)^35^ was used to measure social functioning in schizophrenia participants and controls.

### The SID Task

The SID task^16^, ^17^ was used to measures social motivation. Details of the SID task has been described elsewhere.^17^ Unlike the MID task which uses monetary gain or loss as outcomes, the SID task uses cartoon‐based facial expressions with body gestures (emoji) as social outcomes (e.g. praise or rejection). In brief, the SID task contained 60 trials. Each trial comprised the following phases, namely: (i) cue presentation; (ii) reward anticipation (and delay); (iii) target presentation; (iv) response window and (approach/avoidance) action; (v) feedback; and (vi) outcome phases. In each trial, participants first viewed a cue (either positive, neutral or negative) which indicated three potential social outcomes (reward/praise, neutral or punishment/ rejection). Then, the task had a short delay, during which reward anticipation occurred in participants. After the delay, a target appeared on the screen, and participants had to press the button as soon as possible to hit the target, within a personalized ‘response window’ (see below). Immediate feedback regarding hit or miss would be given, followed by outcome presentation, i.e. cartoon‐pictures of facial expressions (praise/neutral/rejection) and body gestures (e.g. thumbs‐up/thumbs‐down/no‐gesture) in the outcome phase of the trial.

**Fig. 1:**
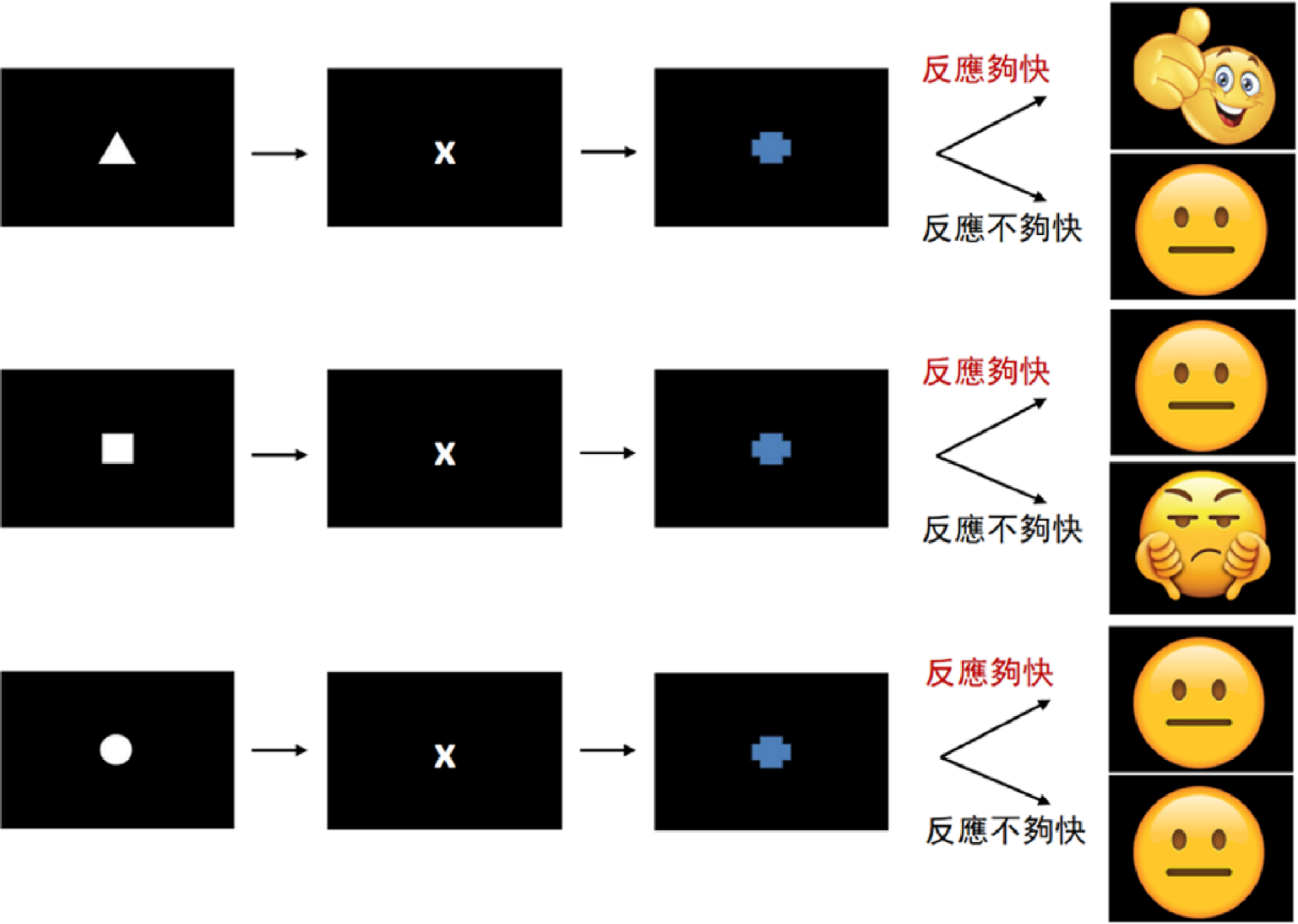
Diagrammatic illustration of the Social Incentive Delay (SID) task. In each trial, a cue indicating the condition (a triangle/square/circle) would be followed by a delayed period (a white ‘x’). Then a target (a blue fixation cross) would appear on the screen, and participants have to press a button as quickly as possible. A personalized response window applies to each participant, based on the results of a simple reaction time task, such as the mean hit rate in the neutral‐cue condition is set as 66%. Based on the participant's response (hit or missed) and the cue conditions, the computer shows the cartoon‐based social reward or punishment. 反應夠快 = hit; 反應不夠快 = missed.

During the reward anticipation phase and the outcome phase, participants were asked to self‐rate their anticipatory and consummatory pleasure on a 9‐point Likert scale shown on the computer screen. In the SID task, each condition (positive, neutral, or negative cue) contained 20 trials, which were presented sequentially in a pseudo‐random manner. The paradigm is illustrated in Fig. 1.

Before the formal SID task, we administered a simple reaction time task to participants and estimated each participant's individual reaction time. We then adjusted the ‘response window’ for neutral targets on a participant‐by‐participant basis, such that the hit rate for the *neutral* condition was set as approximately 66%. Because of the personalized response window for the neutral condition, the hit rate of the SID task is regarded as ‘neutral‐cue‐centered’. However, the hit rate for *reward* and *punishment* conditions could vary differently among participants. The computerized SID task recorded the hit‐or‐not response, reaction time (in ms), anticipatory pleasure and consummatory pleasure ratings, the number of trials (ranged from 1 to 60), and cue/reward condition for each trial. If the participant missed the target, the reaction time would be arbitrarily recorded as 0.

### Data analysis

Statistical analyses were performed using the Statistical Package for the Social Sciences (SPSS) version 21.0 for Windows, and the software R version 4.0.3. The significance was set at *P* < 0.05 (two‐tailed) unless otherwise specified. ANOVAs were used to compare the three groups in age, education, non‐verbal IQ and HAM‐D. We then compared TRS patients and remitted schizophrenia patients in clinical symptoms, number of previous psychiatric admissions, antipsychotic medications, anti‐cholinergic burden, mediation adherence, extra‐pyramidal side‐effects and SOFAS, using independent sample *t*‐tests.

Although previous research on SID used mixed ANOVA models for data analysis,^17^ this traditional method had limitations, such as violation of normality assumption, and potential confounds of practice effect as number of SID trials increased. In this study, we conducted generalized estimation equation (GEE) models^36^ to address the abovementioned limitations. The GEE models examined the impact of the clinical status of TRS and remitted schizophrenia, as well as the social reward/punishment on the SID performances (i.e. hit‐or‐not, anticipatory pleasure and consummatory pleasure). Specifically, we entered the binary variables of positive cue (social reward), negative cue (social punishment), TRS, remitted schizophrenia as the independent variables, and hit‐or‐not as the outcome variable in Model 1a, with the subject number as the cluster variable, and sex, age, non‐verbal IQ, anticholinergic burden, antipsychotic dosage (DDD), and trial number as covariates. Moreover, to examine the interaction between group and valence of cue, we computed four additional independent variables, i.e. TRS × social reward, TRS × social punishment, remitted schizophrenia × social reward, and remitted schizophrenia × social punishment, and entered these four variables into the same GEE model to form Model 1b. Likewise, Models 2a and 2b were built using the same set of independent variables, cluster variable and covariates, except that the outcome variable became anticipatory pleasure. Lastly, in Models 3a and 3b, consummatory pleasure was entered as the outcome variable. All the GEE models used exchangeable correlation structure. We used binomial distribution to model hit‐or‐not; and Poisson distribution to model anticipatory and consummatory pleasure. We did not build any GEE model for reaction time, because non‐hit trials lacked accurate estimates for the variable.

To account for the effects of negative and other clinical symptoms on social motivation processing, we modified the above six GEE models and only used our clinical participants as the sample. Specifically, we computed the binary variable of TRS status by taking the remitted schizophrenia group status as the reference and included the PANSS‐positive symptoms, PANSS‐general symptoms, CAINS‐MAP, and CAIN‐EXP as the additional covariates.

To account for the effects of active psychiatric symptoms of TRS patients on social motivation processing, we categorized our TRS participants into the ‘remitted‐TRS’ group and the ‘non‐remitted’ TRS group.^23^ Specifically, we computed the binary variables of ‘remitted‐TRS’ status and ‘non‐remitted (symptomatic) TRS’ status by taking the control group status as the reference. We repeated the GEE modeling using the same set of independent variables, cluster variable, covariates, and outcome variable as in Models 1–3, but only included our TRS patients and control participants as the sample.

In supplementary analyses, we directly compared the ‘non‐remitted (symptomatic) TRS’ group with the ‘remitted TRS’ group using GEE models, with the ‘remitted‐TRS’ group as the reference (see Supplementary Tables S1–S3). We also examined the correlations of negative symptoms with SID performances in TRS patients and remitted schizophrenia patients separately (see Supplementary Tables S4–S6).

## Results

Table 1 shows the sample characteristics. Among the TRS groups, 12, 28, and 16 participants had ‘early‐emerged TRS’ (<1 year after schizophrenia onset), ‘medium‐emerged TRS’ (2–5 years), and ‘late‐emerged TRS’ (>5 years) respectively^1^; whilst the remaining four participants' onset of TRS could not be clearly ascertained. The TRS, remitted schizophrenia, and control groups were matched in age, sex ratio, and education (*p*s >0.05). However, TRS (*P* = 0.001) and remitted schizophrenia (*P* = 0.031) participants had lower non‐verbal IQ than controls. All participants scored <7 in the 17‐item HAM‐D, indicating absence of depression.^32^ As expected, TRS participants had longer illness duration (mean = 96.3 months, SD = 49.95) than remitted schizophrenia participants (mean = 62.03 months, SD = 55.32) (*t* = 3.561, *P* = 0.001). All schizophrenia patients were taking antipsychotic medications, and all except 5 TRS participants were receiving clozapine. The antipsychotic dosages (DDD) between the TRS and remitted schizophrenia groups were comparable (*t* = 0.218, *P* = 0.828). Moreover, TRS participants had significantly higher anticholinergic medication burden than remitted schizophrenia participants (*t* = 4.979, *P* = 0.001). On the other hand, TRS and remitted schizophrenia participants also showed comparable drug compliance, as reflected in the DAI (*t* = 0.997, *P* = 0.321) and the MARS (*t* = 0.604, *P* = 0.547). Besides, the two schizophrenia groups both exhibited minimal levels of EPSE, as reflected in the ESRS (*t* = −0.509, *P* = 0.612) and the AIMS (*t* = 1.295, *P* = 0.198).

**Table 1:** Demographic and clinical information of the participants

	TRS patients (*N* = 60)		Remitted SCZ (*N* = 60)		Controls (*N* = 60)		F_[2177]_ /t_(118)_/*χ* ^2^	*P*	Post‐hoc (Sidak) pairwise comparison		
	Mean	SD	Mean	SD	Mean	SD			TRS *vs*. Remitted SCZ (Cohen's d)	TRS *vs*. Controls (Cohen's d)	Remitted SCZ *vs*. Controls (Cohen's d)
Age (years)	31.13	6.93	31.53	8.15	31.80	8.06	0.113	0.893			
Sex (male *vs*. female)	34 *vs*. 26		22 *vs*. 38		30 *vs*. 30		4.988*	0.083			
Education (years)	13.03	3.11	13.00	2.55	13.52	2.89	0.613	0.543			
TONI nonverbal IQ	94.20	9.22	96.38	8.89	100.88	10.39	7.684	**0.001**	0.509 (−0.241)	**0.001** (−0.680)	**0.031** (−0.465)
Duration of treatment (months)	77.45	43.20	42.25	40.64			4.597	**<0.001**			
Illness duration months	96.30	49.95	62.03	55.32			3.561	**0.001**			
DDD (mg)	15.44	11.23	15.06	7.38			0.218	0.828			
Anticholinergic burden	3.70	1.23	2.42	1.58			4.979	**0.001**			
Number of psych admissions	1.58	1.24	0.90	0.93			3.412	**<0.001**			
PANSS positive	9.57	3.85	7.60	1.43			3.709	**<0.001**			
PANSS negative	14.95	6.57	11.28	4.79			3.492	**0.001**			
PANSS general	19.20	3.95	18.03	2.21			1.998	**0.048**			
CAINS MAP	16.70	8.64	10.48	6.14			4.545	**<0.001**			
CAINS EXP	7.35	4.24	3.12	3.54			5.934	**<0.001**			
DAI total score	13.20	2.44	12.77	2.32			0.997	0.321			
MARS composite score	7.37	1.33	7.23	1.39			0.604	0.547			
HAMD total	0.65	1.42	0.57	1.14	0.18	0.83	2.771	0.065			
ESRS total	1.32	2.44	1.53	2.22			−0.509	0.612			
AIMS total	0.18	0.60	0.07	0.36			1.295	0.198			
SOFAS	60.85	15.48	66.48	14.05	90.28	2.436	99.192	**<0.001**	**0.035** (−0.381)	**<0.001** (−2.657)	**<0.001** (−2.360)

AIMS, the Abnormal Involuntary Movement Scale; CAINS, the Clinical Assessment Interview for Negative Symptoms; DAI, the Drug Attitude Inventory; DDD, daily defined dose; ESRS, the Extrapyramidal Side‐effect Rating Scale; HAM‐D, the 17‐item Hamilton Rating Scale for Depression; MARS, the Medication Adherence Rating Scale; PANSS, the Positive and Negative Symptom Scales; SCZ, schizophrenia; SOFAS, the Social and Occupational Functioning Assessment Scale; TRS, treatment‐resistant schizophrenia.

* *χ* ^2^ test; significant *P*‐values are bold.

Similar to our previous results,^7^ TRS participants showed higher levels of negative symptoms than remitted schizophrenia participants in CAINS‐MAP (*t* = 4.545, *P* < 0.001) and CAINS‐EXP (*t* = 5.934, *P* < 0.001). TRS participants also showed higher levels of PANSS‐positive (*t* = 3.709, *P* < 0.001) and general (*t* = 3.492, *P* = 0.001) symptoms than remitted schizophrenia patients. Among our TRS participants, 37 of them met the Andreasen's symptom remission criteria,^23^ and therefore were deemed clozapine‐responsive. As expected, the three groups differed in SOFAS (F[2177] = 99.192, *P* < 0.001), with TRS participants having significantly poorer social functioning than remitted schizophrenia participants (*P* = 0.035), whilst remitted schizophrenia participants having poorer social functioning than controls (*P* < 0.001).

### Social motivation

Table 2 shows the performance variables of SID task. The GEE model results for the entire sample are shown in Tables 3, 4, 5. In the GEE models, neutral cue was the reference to positive cue (social reward) and negative cue (social punishment); the control group served as the reference to the TRS group status and remitted schizophrenia group status. In Model 1a, we found that application of negative cues promoted SID accuracy (*β* = 0.091, *P* = 0.003), and male sex was associated with lower SID accuracy (*β* = −0.164, *P* = 0.005). As trial number increased, the SID accuracy improved (*β* = 0.023, *P* < 0.001), suggesting practice effects. However, the TRS status, remitted schizophrenia status, and interactions between group status and cue valence (see Model 1b) all failed to have significant impact on SID accuracy. In Models 2a and 2b, we found that the interaction between TRS and social punishment had resulted in lower anticipatory pleasure (*β* = −0.028, *P* = 0.013). Moreover, the interaction between TRS and social reward showed significant impacts to reduce anticipatory pleasure (*β* = −0.032, *P* = 0.008). In control participants, the anticipated avoidance of social punishment (negative cue) and the anticipated social reward (positive cue) both resulted in an increase of anticipatory pleasure. The negative *β*‐values indicated that such phenomenon was diminished in TRS participants. In Model 3a, we found that the application of negative cues reduced consummatory pleasure (*β* = −0.014, *P* = 0.004), but positive cues promoted consummatory pleasure (*β* = 0.080, *P* < 0.001). As trial number increased, the consummatory pleasure increased (*β* = 0.001, *P* < 0.001). In Model 3b, the interaction between TRS and positive cue had significant impacts on reducing consummatory pleasure (*β* = −0.094, *P* < 0.001). In control participants, the successful avoidance of social punishment (negative cues) and the receipt of social reward (positive cues) both resulted in an increase of consummatory pleasure. The negative *β*‐value indicated that such phenomenon was diminished in TRS participants.

**Table 2:** The mean and SD of performance variables of the Social Incentive Delay (SID) task across participant groups

	TRS patients (N = 60)		Remitted SCZ patients (N = 60)		Controls (N = 60)	
	Mean	SD	Mean	SD	Mean	SD
Accuracy						
Positive cue	0.596	0.120	0.605	0.116	0.632	0.066
Neutral cue	0.602	0.132	0.604	0.131	0.638	0.065
Negative cue	0.594	0.114	0.611	0.110	0.645	0.056
Reaction time (ms)						
Positive cue	133.838	41.933	141.027	45.402	133.291	22.990
Neutral cue	137.403	41.950	143.328	45.492	135.932	27.308
Negative cue	135.683	40.227	143.255	41.740	139.218	27.838
Anticipatory pleasure						
Positive cue	5.288	1.208	5.146	1.044	5.600	0.880
Neutral cue	5.348	1.216	5.094	1.042	5.478	0.844
Negative cue	5.240	1.213	5.123	1.008	5.528	0.922
Consummatory pleasure						
Positive cue	5.567	1.167	5.788	1.105	6.069	0.842
Neutral cue	5.403	1.207	5.248	0.988	5.275	0.762
Negative cue	5.243	1.257	5.195	1.034	5.128	0.839

SCZ, schizophrenia; TRS, treatment‐resistant schizophrenia.

**Table 3:** Generalized Estimation Equation (GEE) model results for SID hit‐or‐not

	beta‐coefficient	SE	*P*‐value
Model 1a (all participants)			
Subject	8.86E‐05	8.52E‐05	0.299
Negative cue	9.08E‐02	3.04E‐02	**0.003**
Positive Cue	−2.68E‐02	2.93E‐02	0.360
Remitted SCZ	−1.37E‐01	1.64E‐01	0.403
TRS	−2.85E‐01	2.08E‐01	0.170
Sex (male)	−1.64E‐01	5.78E‐02	**0.005**
Age	9.57E‐04	3.59E‐03	0.606
Trial number	2.26E‐02	1.97E‐03	**<0.001**
TONI nonverbal IQ	4.53E‐03	2.62E‐03	0.083
Anticholinergic burden	4.64E‐02	3.93E‐02	0.238
DDD (mg)	2.39E‐03	4.64E‐03	0.606
Model 1b (all participants)			
Subject	8.88E‐05	8.52E‐05	0.297
Negative cue	1.58E‐01	5.41E‐02	**0.004**
Positive Cue	−3.31E‐02	5.02E‐02	0.509
Remitted SCZ	−1.25E‐01	1.73E‐01	0.472
TRS	−2.41E‐01	2.15E‐01	0.263
Sex (male)	−1.64E‐01	5.78E‐02	**0.005**
Age	9.48E‐04	3.60E‐03	0.792
Trial number	2.26E‐02	1.98E‐03	**<0.001**
TONI nonverbal IQ	4.52E‐03	2.62E‐03	0.084
Anticholinergic burden	4.65E‐02	3.93E‐02	0.237
DDD (mg)	2.38E‐03	4.64E‐03	0.607
Remitted SCZ × Negative Cue	−6.33E‐02	7.29E‐02	0.386
Remitted SCZ × Positive Cue	2.57E‐02	7.15E‐02	0.719
TRS × Negative cue	−1.27E‐01	7.44E‐02	0.089
TRS × Positive Cue	7.93E‐03	7.12E‐02	0.911

Significant *P*‐values are bold.

DDD, daily defined dose of antipsychotics; SCZ, schizophrenia; TRS, treatment‐resistant schizophrenia.

**Table 4:** Generalized Estimation Equation (GEE) model results for anticipatory pleasure

	beta‐coefficient	SE	*P*‐value
Model 2a (all participants)			
Subject	2.90E‐05	3.41E‐05	0.396
Negative cue	−1.58E‐03	4.05E‐03	0.697
Positive Cue	7.01E‐03	5.42E‐03	0.196
Remitted SCZ	−3.78E‐02	7.60E‐02	0.619
TRS	1.17E‐02	6.98E‐02	0.867
Sex (male)	−5.09E‐03	3.18E‐02	0.873
Age	8.05E‐05	1.52E‐03	0.958
Trial number	2.16E‐04	2.97E‐04	0.468
TONI nonverbal IQ	−6.96E‐04	1.16E‐03	0.547
Anticholinergic burden	−1.57E‐02	1.27E‐02	0.214
DDD (mg)	2.83E‐03	2.42E‐03	0.242
Model 2b (all participants)			
Subject	2.96E‐05	3.39E‐05	0.383
Negative cue	9.99E‐03	7.89E‐03	0.205
Positive Cue	2.33E‐02	7.89E‐03	**0.025**
Remitted SCZ	−2.91E‐02	7.68E‐02	0.705
TRS	3.31E‐02	7.15E‐02	0.644
Sex (male)	−6.74E‐03	3.17E‐02	0.832
Age	−2.38E‐05	1.52E‐03	0.988
Trial number	2.17E‐04	2.97E‐04	0.466
TONI nonverbal IQ	−7.03E‐04	1.15E‐03	0.541
Anticholinergic burden	−1.59E‐02	1.27E‐02	0.211
DDD (mg)	2.83E‐03	2.40E‐03	0.238
Remitted SCZ × Negative Cue	−5.41E‐03	1.03E‐02	0.601
Remitted SCZ × Positive Cue	−1.51E‐02	1.51E‐02	0.319
TRS × Negative cue	−2.76E‐02	1.11E‐02	**0.013**
TRS × Positive Cue	−3.16E‐02	1.19E‐02	**0.008**

Significant *P*‐values are bold.

DDD, daily‐defined dose of antipsychotics; SCZ, schizophrenia; TRS, treatment‐resistant schizophrenia.

**Table 5:** Generalized Estimation Equation (GEE) model results for consummatory pleasure

	beta‐coefficient	SE	*P*‐value
Model 3a (all participants)			
Subject	5.11E‐05	3.32E‐05	0.124
Negative cue	−1.42E‐02	4.91E‐03	**0.004**
Positive Cue	8.00E‐02	1.00E‐02	**<0.001**
Remitted SCZ	2.01E‐03	6.62E‐02	0.976
TRS	−2.05E‐02	6.29E‐02	0.744
Sex (male)	1.35E‐03	2.76E‐02	0.961
Age	−2.19E‐04	1.36E‐03	0.872
Trial number	1.34E‐03	3.13E‐04	**<0.001**
TONI nonverbal IQ	−5.43E‐04	1.28E‐03	0.672
Anticholinergic burden	4.70E‐03	1.14E‐02	0.68
DDD (mg)	1.89E‐03	2.05E‐03	0.358
Model 3b (all participants)			
Subject	4.98E‐05	3.34E‐05	0.136
Negative cue	−1.20E‐02	9.15E‐03	0.191
Positive Cue	1.24E‐01	2.04E‐02	**<0.001**
Remitted SCZ	1.71E‐02	6.82E‐02	0.802
TRS	3.47E‐02	6.40E‐02	0.587
Sex (male)	3.24E‐03	2.79E‐02	0.908
Age	−4.54E‐04	1.37E‐03	0.741
Trial number	1.34E‐03	3.13E‐04	**<0.001**
TONI nonverbal IQ	−5.76E‐04	1.30E‐03	0.657
Anticholinergic burden	4.51E‐03	1.13E‐02	0.691
DDD (mg)	2.03E‐03	2.08E‐03	0.329
Remitted SCZ × Negative Cue	5.20E‐03	1.28E‐02	0.684
Remitted SCZ × Positive Cue	−3.26E‐02	2.60E‐02	0.211
TRS × Negative cue	−1.13E‐02	1.22E‐02	0.356
TRS × Positive Cue	−9.41E‐02	2.42E‐02	**<0.001**

Significant *P*‐values are bold.

DDD, daily‐defined dose of antipsychotics; SCZ, schizophrenia; TRS, treatment‐resistant schizophrenia.

The GEE model results for the clinical sample evaluated the impacts of negative and other symptoms on social motivation processing in TRS patients (see Tables 6, 7, 8). In Model 4a, we found higher CAINS‐EXP was associated with lower SID accuracy (*β* = −0.037, *P* = 0.006) in TRS and remitted schizophrenia participants, suggesting that negative symptoms could affect social motivation processing. As expected, interactions between TRS group status and cue valence (see Model 4b) did not show any significant impact on SID accuracy, after controlling for the CAINS‐MAP and CAINS‐EXP. In Models 5a and 6a, the CAINS‐MAP and CAINS‐EXP did not show any significant impacts on TRS and remitted schizophrenia participants' anticipatory pleasure and consummatory pleasure in the SID task (*p*s > 0.05). As shown in Model 5b, the interaction between TRS and social punishment remained significant, even after controlling for the PANSS‐positive symptoms, PANSS‐general symptoms, and CAINS negative symptoms (*β* = −0.022, *P* = 0.026). In remitted schizophrenia participants (the reference group), the anticipated avoidance of social punishment (negative cue) resulted in reduction of anticipatory pleasure. The negative *β*‐values indicated that such phenomenon was diminished in TRS participants, relative to remitted schizophrenia participants. Lastly, Model 6b showed that the interaction between TRS and social reward remained significant, even after controlling for the PANSS‐positive symptoms, PANSS‐general symptoms, and CAINS negative symptoms (*β* = −0.062, *P* = 0.003). In remitted schizophrenia participants (the reference group), the receipt of social reward (positive cue) resulted in higher consummatory pleasure. The negative *β*‐values indicated that such phenomenon was diminished in TRS participants, relative to remitted schizophrenia participants.

**Table 6:** Generalized Estimation Equation (GEE) model results for SID hit‐or‐not (with clinical symptoms as covariates)

	beta‐coefficient	SE	*P*‐value
Model 4a (TRS patients + remitted SCZ patients)			
Subject	3.46E‐05	1.01E‐04	0.731
Negative cue	6.50E‐02	3.64E‐02	0.074
Positive Cue	−2.46E‐02	3.62E‐02	0.498
TRS	−7.13E‐02	8.73E‐02	0.414
Sex (male)	−2.75E‐01	7.53E‐02	**<0.001**
Age	3.36E‐03	4.80E‐03	0.484
Trial number	2.32E‐02	2.45E‐03	**<0.001**
TONI nonverbal IQ	4.68E‐03	3.41E‐03	0.170
Anticholinergic burden	4.89E‐02	3.40E‐02	0.151
DDD (mg)	4.54E‐03	4.45E‐03	0.308
PANSS‐positive	−1.57E‐03	1.31E‐02	0.904
PANSS‐general	−2.27E‐02	1.75E‐02	0.193
CAINS‐MAP	1.40E‐02	7.69E‐03	0.070
CAINS‐EXP	−3.67E‐02	1.33E‐02	**0.006**
Model 4b (TRS patients + remitted SCZ patients)			
Subject	3.46E‐05	1.01E‐04	0.731
Negative cue	9.69E‐02	5.04E‐02	0.055
Positive Cue	−7.57E‐03	5.14E‐02	0.883
TRS	−3.91E‐02	1.00E‐01	0.697
Sex (male)	−2.75E‐01	7.53E‐02	**<0.001**
Age	3.35E‐03	4.81E‐03	0.485
Trial number	2.32E‐02	2.45e‐03	**<0.001**
TONI nonverbal IQ	4.67E‐03	3.41E‐03	0.170
Anticholinergic burden	4.90E‐02	3.40E‐02	0.150
DDD (mg)	4.54E‐03	4.45E‐03	0.308
PANSS‐positive	−1.57E‐03	1.31E‐02	0.905
PANSS‐general	−2.27E‐02	1.75E‐02	0.193
CAINS‐MAP	1.40E‐02	7.70E‐03	0.070
CAINS‐EXP	−3.66E‐02	1.33E‐02	**0.006**
TRS × Negative cue	−6.39E‐02	7.22E‐02	0.376
TRS × Positive Cue	−3.42E‐02	7.24E‐02	0.637

Significant *P*‐values are bold.

CAINS, the Clinical Assessment Interview for Negative Symptoms; DDD, daily‐defined dose; PANSS, the Positive and Negative Symptom Scales; SCZ, schizophrenia; TRS, treatment‐resistant schizophrenia.

**Table 7:** Generalized Estimation Equation (GEE) model results for anticipatory pleasure (with clinical symptoms as covariates)

	beta‐coefficient	SE	*P*‐value
Model 5a (TRS patients + remitted SCZ patients)			
Subject	7.78E‐05	4.73E‐05	0.100
Negative cue	−6.69E‐03	4.76E‐03	0.160
Positive Cue	−1.71E‐04	6.19E‐03	0.978
TRS	3.95E‐02	5.56E‐02	0.477
Sex (male)	1.12E‐02	4.11E‐02	0.785
Age	−1.45E‐03	2.13E‐03	0.498
Trial number	2.00E‐04	3.66E‐04	0.584
TONI nonverbal IQ	−5.24E‐04	1.47E‐03	0.721
Anticholinergic burden	−1.35E‐02	1.37E‐02	0.325
DDD (mg)	2.28E‐03	2.45E‐03	0.351
PANSS‐positive	1.10E‐02	7.25E‐03	0.127
PANSS‐general	−5.69E‐03	6.53E‐03	0.384
CAINS‐MAP	−2.37E‐03	3.30E‐03	0.473
CAINS‐EXP	−7.33E‐06	6.59E‐03	0.999
Model 5b (TRS patients + remitted SCZ patients)			
Subject	7.79E‐05	4.73E‐05	0.099
Negative cue	4.54E‐03	6.25E‐03	0.468
Positive Cue	8.22E‐03	1.10E‐02	0.453
TRS	5.23E‐02	5.65E‐02	0.354
Sex (male)	−1.23E‐02	4.09E‐02	0.763
Age	−1.50E‐03	2.12E‐03	0.481
Trial number	2.01E‐04	3.66E‐04	0.583
TONI nonverbal IQ	−5.21E‐04	1.46E‐03	0.721
Anticholinergic burden	−1.38E‐02	1.37E‐02	0.312
DDD (mg)	2.28E‐03	2.44E‐03	0.350
PANSS‐positive	1.12E‐02	7.20E‐03	0.122
PANSS‐general	−5.59E‐03	6.51E‐03	0.391
CAINS‐MAP	−2.30E‐03	3.29E‐03	0.485
CAINS‐EXP	−3.39E‐04	6.59E‐03	0.959
TRS × Negative cue	−2.22E‐02	9.99E‐03	**0.026**
TRS × Positive Cue	−1.65E‐02	1.24E‐02	0.182

Significant *P*‐values are bold.

CAINS, the Clinical Assessment Interview for Negative Symptoms; DDD, daily‐defined dose; PANSS, the Positive and Negative Symptom Scales; SCZ, schizophrenia; TRS, treatment‐resistant schizophrenia.

**Table 8:** Generalized Estimation Equation (GEE) model results for consummatory pleasure (with clinical symptoms as covariates)

	beta‐coefficient	SE	*P*‐value
Model 6a (TRS patients + remitted SCZ patients)			
Subject	1.16E‐04	4.64E‐05	**0.013**
Negative cue	−1.48E‐02	5.87E‐03	**0.012**
Positive Cue	6.10E‐02	1.08E‐02	**<0.001**
TRS	−1.45E‐02	4.97E‐02	0.770
Sex (male)	−2.26E‐02	3.42E‐02	0.508
Age	−1.40E‐03	1.76E‐03	0.427
Trial number	1.43E‐03	4.17E‐04	**<0.001**
TONI nonverbal IQ	−4.91E‐05	1.72E‐03	0.977
Anticholinergic burden	8.34E‐03	1.20E‐02	0.486
DDD (mg)	1.87E‐03	2.00E‐03	0.351
PANSS‐positive	8.93E‐03	6.73E‐03	0.185
PANSS‐general	−7.54E‐03	7.66E‐03	0.325
CAINS‐MAP	−4.78E‐03	3.74E‐03	0.202
CAINS‐EXP	−2.92E‐04	7.02E‐03	0.967
Model 6b (TRS patients + remitted SCZ patients)			
Subject	1.18E‐04	4.64E‐05	**0.011**
Negative cue	−6.52E‐03	8.92E‐03	0.465
Positive Cue	9.17E‐02	1.63E‐02	**<0.001**
TRS	2.41E‐02	5.12E‐02	0.638
Sex (male)	−2.47E‐02	3.45E‐02	0.474
Age	−1.67E‐03	1.77E‐03	0.346
Trial number	1.44E‐03	4.16E‐04	**0.001**
TONI nonverbal IQ	−3.90E‐05	1.74E‐03	0.982
Anticholinergic burden	7.81E‐03	1.19E‐02	0.512
DDD (mg)	1.90E‐03	2.01E‐03	0.345
PANSS‐positive	9.53E‐03	6.74E‐03	0.157
PANSS‐general	−7.52E‐03	7.65E‐03	0.326
CAINS‐MAP	−5.20E‐03	3.76E‐03	0.166
CAINS‐EXP	1.37E‐05	7.07E‐03	0.998
TRS × Negative cue	−1.64E‐02	1.20E‐02	0.171
TRS × Positive Cue	−6.18E‐02	2.08E‐02	**0.003**

Significant *P*‐values are bold.

CAINS, the Clinical Assessment Interview for Negative Symptoms; DDD, daily‐defined dose; PANSS, the Positive and Negative Symptom Scales; SCZ, schizophrenia; TRS, treatment‐resistant schizophrenia.

The GEE model results with the differentiation between the remitted‐TRS and non‐remitted (symptomatic) TRS status further clarified the impacts of symptom remission on social motivation processing in TRS patients (see Tables 9, 10, 11). In Model 7b, the remitted‐TRS status, non‐remitted (symptomatic) TRS status, and interactions between the TRS group status and cue valence all failed to have a significant impact on SID accuracy, consistent with our earlier GEE model results. In Model 8b, we found that the interaction between remitted‐TRS status and social punishment still showed significant impacts to reduce anticipatory pleasure (*β* = −0.042, *P* = 0.013), and the interaction between remitted‐TRS status and social reward also still resulted in lower anticipatory pleasure (*β* = −0.039, *P* = 0.019), albeit the limited sample size of the remitted‐TRS group (n = 37). In control participants, the anticipated avoidance of social punishment (negative cue) and the anticipated social reward (positive cue) both resulted in an increase of anticipatory pleasure. The negative *β*‐values indicated that such phenomenon was diminished in remitted‐TRS participants. Regarding the impacts of non‐remitted (symptomatic) TRS status on social motivation processing, the interaction between non‐remitted‐TRS status and social reward had significantly reduced anticipatory pleasure (*β* = −0.028, *P* = 0.016). However, the interaction between non‐remitted (symptomatic) TRS status and social reward failed to have significant impacts on anticipatory pleasure (*P* > 0.05), likely related to the limited sample size of the non‐remitted (symptomatic) TRS group (n = 23). In Model 9b, we found that both the interaction between remitted‐TRS status and positive cue (*β* = −0.110, *P* = 0.002), and the interaction between non‐remitted (symptomatic) TRS status and positive cue (*β* = −0.085, *P* < 0.001) remained having significant impacts on reducing consummatory pleasure, albeit the limited sample size. In control participants, the successful avoidance of social punishment (negative cues) and the receipt of social reward (positive cues) both resulted in an increase of pleasure. The negative *β*‐value indicated that such phenomenon was diminished in both remitted‐TRS and non‐remitted (symptomatic) TRS participants.

**Table 9:** Generalized Estimation Equation (GEE) model results for SID hit‐or‐not (with differentiation between remitted‐TRS and non‐remitted TRS status)

	beta‐coefficient	SE	*P*‐value
Model 7a (Remitted TRS patients + non‐remitted TRS patients + controls)			
Subject	5.00E‐05	1.10E‐04	0.648
Negative cue	8.50E‐02	3.79E‐02	**0.025**
Positive Cue	−3.68E‐02	3.56E‐02	0.301
Remitted TRS	−3.41E‐01	4.02E‐01	0.397
Non‐remitted TRS	−2.28E‐01	4.27E‐01	0.594
Sex (male)	−1.62E‐01	7.71E‐02	**0.035**
Age	−1.38E‐03	4.38E‐03	0.753
Trial number	2.14E‐02	2.39E‐03	**<0.001**
TONI nonverbal IQ	5.20E‐03	2.96E‐03	0.079
Anticholinergic burden	2.68E‐02	9.74E‐02	0.783
DDD (mg)	3.51E‐03	6.54E‐03	0.591
Model 7b (Remitted TRS patients + non‐remitted TRS patients + controls)			
Subject	5.05E‐05	1.10E‐04	0.645
Negative cue	1.54E‐01	5.42E‐02	**0.004**
Positive cue	−3.27E‐02	5.01E‐02	0.514
Remitted TRS	−2.66E‐01	4.16E‐01	0.522
Non‐remitted TRS	−2.01E‐01	4.28E‐01	0.638
Sex (male)	−1.63E‐01	7.71E‐02	**0.035**
Age	−1.38E‐03	4.39E‐03	0.754
Trial number	2.14E‐02	2.40E‐03	**<0.001**
TONI nonverbal IQ	5.19E‐03	2.96E‐03	0.079
Anticholinergic burden	2.67E‐02	9.75E‐02	0.784
DDD (mg)	3.51E‐03	6.53E‐03	0.592
Remitted TRS × Negative Cue	−1.15E‐01	1.04E‐01	0.270
Remitted TRS × Positive Cue	−1.07E‐01	9.87E‐02	0.277
Non‐remitted TRS × Negative cue	−1.32E‐01	8.16E‐02	0.105
Non‐remitted TRS × Positive Cue	5.36E‐02	7.85E‐02	0.495

Significant *P*‐values are bold.

CAINS, the Clinical Assessment Interview for Negative Symptoms; DDD, daily‐defined dose; PANSS, the Positive and Negative Symptom Scales; TRS, treatment‐resistant schizophrenia.

**Table 10:** Generalized Estimation Equation (GEE) model results for anticipatory pleasure (with differentiation between remitted‐TRS and non‐remitted TRS status)

	beta‐coefficient	SE	*P*‐value
Model 8a (Remitted TRS patients + non‐remitted TRS patients + controls)			
Subject	4.96E‐05	5.00E‐05	0.322
Negative cue	−3.51E‐03	5.30E‐03	0.507
Positive Cue	6.27E‐03	5.97E‐03	0.293
Remitted TRS	1.72E‐01	1.23E‐01	0.161
Non‐remitted TRS	1.48E‐01	9.89E‐02	0.135
Sex (male)	1.59E‐02	3.99E‐02	0.692
Age	2.15E‐04	2.14E‐03	0.920
Trial number	6.45E‐04	3.63E‐04	0.075
TONI nonverbal IQ	−1.46E‐03	1.50E‐03	0.331
Anticholinergic burden	−5.37E‐02	2.33E‐02	**0.021**
DDD (mg)	3.80E‐03	3.34E‐03	0.255
Model 8b (Remitted TRS patients + non‐remitted TRS patients + controls)			
Subject	5.00E+00	4.95E‐05	0.316
Negative cue	1.13E‐02	7.81E‐03	0.147
Positive Cue	2.33E‐02	1.05E‐02	**0.026**
Remitted TRS	1.96E‐01	1.27E‐01	0.123
Non‐remitted TRS	1.62E‐01	1.02E‐01	0.111
Sex (male)	1.29E‐02	3.98E‐02	0.746
Age	3.71E‐06	2.14E‐03	0.999
Trial number	6.47E‐04	3.63E‐04	0.075
TONI nonverbal IQ	−1.49E‐03	1.49E‐03	0.320
Anticholinergic burden	−5.35E‐02	2.40E‐02	**0.026**
DDD (mg)	3.85E‐03	3.32E‐03	0.246
Remitted TRS × Negative Cue	−4.20E‐02	1.68E‐02	**0.013**
Remitted TRS × Positive Cue	−3.88E‐02	1.66E‐02	**0.019**
Non‐remitted TRS × Negative cue	−1.87E‐02	1.15E‐02	0.104
Non‐remitted TRS × Positive Cue	−2.75E‐02	1.14E‐02	**0.016**

Significant *P*‐values are bold.

CAINS, the Clinical Assessment Interview for Negative Symptoms; DDD, daily‐defined dose; PANSS, the Positive and Negative Symptom Scales; TRS, treatment‐resistant schizophrenia.

**Table 11:** Generalized Estimation Equation (GEE) model results for consummatory pleasure (with differentiation between remitted‐TRS and non‐remitted TRS status)

	beta‐coefficient	SE	*P*‐value
Model 9a (Remitted TRS patients + non‐remitted TRS patients + controls)			
Subject	5.64E‐05	4.56E‐05	0.216
Negative cue	−1.72E‐02	5.83E‐03	**0.003**
Positive Cue	7.37E‐02	1.26E‐02	**<0.001**
Remitted TRS	2.46E‐03	1.03E‐01	0.981
Non‐remitted TRS	3.39E‐02	9.09E‐02	0.709
Sex (male)	3.13E‐02	3.39E‐02	0.357
Age	−7.16E‐04	1.86E‐03	0.700
Trial number	1.66E‐03	3.72E‐04	**<0.001**
TONI nonverbal IQ	−1.14E‐03	1.53E‐03	0.457
Anticholinergic burden	2.80E‐04	2.40E‐02	**0.991**
DDD (mg)	4.95E‐04	2.96E‐03	0.867
Model 9b (Remitted TRS patients + non‐remitted TRS patients + controls)			
Subject	5.42E‐05	4.59E‐05	0.237
Negative cue	−1.10E‐02	9.16E‐03	0.230
Positive Cue	1.24E‐01	2.04E‐02	**<0.001**
Remitted TRS	6.53E‐02	1.05E‐01	0.533
Non‐remitted TRS	7.69E‐02	9.20E‐02	0.403
Sex (male)	3.49E‐02	3.47E‐02	0.315
Age	−1.10E‐03	1.88E‐03	0.560
Trial number	1.67E‐03	3.71E‐04	**<0.001**
TONI nonverbal IQ	−1.19E‐03	1.54E‐03	0.441
Anticholinergic burden	1.36E‐04	2.40E‐02	0.996
DDD (mg)	7.57E‐04	3.02E‐03	0.802
Remitted TRS × Negative Cue	−2.67E‐02	1.71E‐02	0.118
Remitted TRS × Positive Cue	−1.10E‐01	3.50E‐02	**0.002**
Non‐remitted TRS × Negative cue	−2.12E‐03	1.30E‐02	0.871
Non‐remitted TRS × Positive Cue	−8.52E‐02	2.36E‐02	**<0.001**

Significant *P*‐values are bold.

CAINS, the Clinical Assessment Interview for Negative Symptoms; DDD, daily‐defined dose; PANSS, the Positive and Negative Symptom Scales; TRS, treatment‐resistant schizophrenia.

## Discussion

Although impaired social motivation has not been regarded as one of the core features for TRS,^37^ some historical constructs related to treatment non‐response, such as hebephrenic schizophrenia and deficit syndrome of schizophrenia,^38^ are characterized by negative symptoms, including social anhedonia. The latest TRRIP criteria^1^ advocates the characterization of TRS patients based on positive, negative and cognitive symptoms, to identify possible intervention targets for improving patients' social functioning and clinical outcomes. This study is among the first few research to characterize the nature and extent of social motivation processing in TRS patients, using an experimental paradigm and GEE models. Although social reward and social punishment in everyday life can be far more complicated than the cartoon images used in the SID task, our behavioral paradigm has tapped into participants' motivation processing, in view of the observed significant association of negative cues with the outcome of ‘hit‐or‐not’, and the significant association of social reward/punishment with consummatory pleasure. Moreover, other social cognitive paradigms^39^, ^40^ have also used cartoon‐based images (e.g. emoji) to present social‐salient information. Therefore, the cartoon‐based images presented in the outcome phase of the SID task are believed to contain social‐relevant information, and could motivate participants' task performance.

Our results suggested that TRS, remitted schizophrenia, and interaction between TRS/remitted schizophrenia and valence of cue did not have significant impacts on SID accuracy. In motivation processing,^3^, ^15^, ^41^ important components include cost–benefit computation, anticipatory and consummatory pleasure, action‐plan formation, and execution of action. Among the three SID performance variables used in our study (i.e. ‘hit‐or‐not’, anticipatory pleasure, consummatory pleasure), the SID accuracy reflected participants' executed action within a short response window, and might not be a sensitive index to discriminate TRS patients and remitted schizophrenia patients from healthy controls. Other behavioral paradigms, such as effort‐based decision‐making task with social scenarios,^15^ may better capture effort‐expenditure with high sensitivity. On the other hand, we found that the anticipated avoidance of social punishment and anticipated social reward could bring significantly less increment of anticipatory pleasure to TRS patients than healthy controls, as evidenced by the significant interaction between TRS group status and valence of cues in the GEE model for anticipatory pleasure. Similarly, the receipt of social reward could bring significantly less increment of consummatory pleasure to TRS patients than healthy controls. Together, these findings indicate that TRS is associated with altered anticipatory pleasure and consummatory pleasure in social motivation processing, relative to healthy people. Our findings also extended the previous research on non‐social motivation deficits in TRS patients^41^ to social motivation processing.

Consistent with our hypothesis, negative symptoms showed a significantly negative impact on social motivation processing in TRS patients and remitted schizophrenia patients, as evidenced by the significant effects of the CAIN‐EXP on SID ‘hit‐or‐not’ outcome in the GEE models. Nevertheless, the CAINS‐MAP and CAIN‐EXP did not appear to influence other SID performance variables, including anticipatory pleasure and consummatory pleasure in TRS and remitted schizophrenia patients. These unexpected results may be related to the fact that the majority (n = 97) of our clinical sample had fulfilled the Andreasen's symptom remission criteria,^23^ and exhibited minimal levels of negative symptoms.

Given that healthy controls were not measured with the CAINS, the GEE models which controlled for the impacts of negative symptoms on SID performance only included clinical participants. After controlling for negative (and other) symptoms, TRS patients still anticipated significantly less pleasure for the anticipated avoidance of social punishment and experienced less consummatory pleasure for the receipt of social reward, relative to remitted schizophrenia patients. Moreover, after differentiating the remitted‐TRS status from the non‐remitted (symptomatic) TRS status, the GEE models showed that remitted‐TRS patients still exhibited altered anticipatory pleasure and consummatory pleasure in social motivation processing, relative to healthy people, as evidenced by the significant interaction between remitted‐TRS group status and valence of cues in the GEE models for anticipatory and consumatory pleasure. Together, such findings support our hypothesis that TRS patients have altered social motivation processing, regardless of remission status and even after controlling for the effects of negative and other symptoms.

Our preliminary findings of altered social motivation processing in TRS patients may have clinical implications. Around 23% of first‐episode schizophrenia patients would eventually develop treatment‐resistance,^42^ but the effectiveness of current evidence‐based treatments for TRS remains unsatisfactory.^43^ Many TRS patients who responded to clozapine continued to have poor functional outcomes.^44^ Our findings lend support to the clinical potentials of intervening social motivation processing to benefit TRS patients' social functioning. Although cognitive remediation has been reported to improve functional outcomes of TRS patients,^45^ such intervention does not target at social motivation. Motivational interviewing coupled with cognitive behavioral therapy^46^ and cognitive‐behavioral social‐skill training^47^ have been proposed as potential interventions for improving social motivation in schizophrenia patients.^10^ Future study should explore the feasibility of applying these strategies to TRS patients, and evaluate its impacts on negative symptoms and social functioning.

Furthermore, our work may have theoretical implications. Interestingly, motivation has been recently reported to be related to treatment engagement and treatment response in schizophrenia patients.^48^ It is plausible that impaired social motivation processing may contribute to the emergence of negative symptoms in schizophrenia patients and may undermine patients' treatment response to antipsychotics. A recent study examined whether early emergence of negative symptoms could predict future development of TRS, but the results were largely non‐significant.^49^ However, such negative findings may be limited by the crude measure of negative symptoms using a first‐generation scale and the lack of experimental paradigms to tap into cognitive and motivation processing closely related to negative symptoms. Future research should further clarify whether social motivation would predict negative symptoms and non‐response to treatments.

Although the ‘liking‐wanting anhedonia paradox’ suggested that schizophrenia patients tended to have intact ‘liking’ but impaired ‘wanting’,^50^ recent meta‐analytic evidence indicated that schizophrenia patients might have diminished ‘social’ consummatory pleasure, relative to healthy people.^51^ Our findings supported alterations of social motivation processing in TRS patients relative to remitted schizophrenia and controls, affecting anticipatory and consummatory pleasure. Such findings appeared to concur with the current notions.^50^, ^51^ On the other hand, remitted schizophrenia patients did not differ in social motivation processing than controls, contrary to prior research using other paradigms for social motivation processing (such as effort‐based decision‐making tasks).^14^, ^15^ The divergent findings might be related to the fact that all remitted schizophrenia participants had fulfilled the Andreasen's symptom remission criteria,^23^ and exhibited minimal levels of symptoms. Prior research on social motivation processing in schizophrenia patients did not recruit samples in full clinical remission.^14^, ^15^ Another possible reason may be related to the limitations of the SID paradigm,^16^, ^17^ which only used cartoon‐based social reward and punishment.

This study has several limitations. Although our TRS participants had shorter illness duration than many previous chronic TRS samples,^7^, ^8^, ^41^, ^44^ they still had a considerable length of illness duration, longer than the remitted schizophrenia participants. Moreover, social isolation had been found to affect social approach motivation,^52^ but it was not measured in this study. Future study may recruit recently‐emerged TRS patients and measure participants' levels of loneliness to account for these confounds. Third, although the two clinical groups had comparable anticholinergic burden, antipsychotic‐induced parkinsonism side‐effects, and depressive symptoms, we did not control for antipsychotic‐induced sedation, which might lead to secondary negative symptoms affecting social motivation processing. Fourth, the SID task is not a sophisticated task to capture social motivation clearly, which might have resulted in the non‐significant impacts of TRS/remitted schizophrenia and the interaction between TRS/remitted schizophrenia and the valence of cue on the SID accuracy. More sophisticated behavioral paradigms, such as the effort‐based decision‐making task with social scenarios,^15^ should be used in future research to tap into social motivation processing. Fifth, this study did not employ a non‐social behavioral task; therefore, we could not make a comparison between social and non‐social motivation processing in TRS patients. Lastly, this study was cross‐sectional, and we could not infer the contribution of social motivation deficits to negative symptoms.

To conclude, using an experimental paradigm and the more sophisticated GEE models, our preliminary findings suggested that TRS patients exhibited impaired social motivation processing, affecting their ability to anticipate and experience pleasure to social reward/punishment. Future research should adopt a more sophisticated behavioral task to clarify whether social motivation deficits in schizophrenia may predict the risk for developing TRS and functional impairments.

## Author contributions

Simon S.Y. Lui: Conceptualization, Formal analysis, Methodology, Supervision, Visualization, Writing – original draft, Writing –review and editing. Jasmine W.S. Chan: Conceptualization, Data curation, Investigation, Formal analysis, Methodology, Project administration, Writing – original draft, Writing –review and editing. Jason L.F. Chan: Data curation, Investigation, Writing – review and editing. Kimi H.Y. Lam: Data curation, Investigation, Writing – review and editing. Lok‐Yin Choi: Formal analysis, Visualization, Writing – review and editing. Raisie W.K. Wong: Data curation, Investigation, Writing – review and editing. Perry B.M. Leung: Validation, Writing – review and editing. Na Zhan: Writing – review and editing. Jason W.Y. Wong: Project administration, Resources. Mindi W.Y. Chiu: Project administration, Methodology, Resources. Jenny P.H. Lam: Project administration, Resources. Raymond C.K. Chan: Conceptualization, Methodology, Supervision, Writing – review and editing.

## Disclosure statement

The authors declare no conflicts of interest with respect to the authorship or the publication of this article.

## Supporting information


**Table S1.** Generalized Estimation Equation (GEE) model results for SID hit‐or‐not to compare remitted TRS with non‐remitted TRS patients.
**Table S2.** Generalized Estimation Equation (GEE) model results for anticipatory pleasure to compare remitted TRS with non‐remitted TRS patients.
**Table S3.** Generalized Estimation Equation (GEE) model results for consummatory pleasure to compare remitted TRS with non‐remitted TRS patients.
**Table S4.** Spearman's correlations of change in mean scores of SID performance with the CAINS subscale scores in the clinical sample (n = 120).
**Table S5.** Spearman's correlations of change in mean SID anticipatory pleasure ratings with the CAINS subscale scores in TRS sample (n = 60).
**Table S6.** Spearman's correlations of change in mean SID anticipatory pleasure ratings with the CAINS subscale scores in remitted schizophrenia sample (n = 60).

## Data Availability

The data that support the findings of this study are available on request from the corresponding author. The data are not publicly available due to privacy or ethical restrictions.
