# Supplementary material for: Characterizing social motivation deficits in treatment‐resistant schizophrenia: Evidence from social incentive performance
Source: Psychiatry Clin Neurosci. 2026 Jun 17;80(9):773–84. doi: 10.1111/pcn.70094 (PMC13537369; doi:10.1111/pcn.70094)
Supplement: Supplementary file 1 [file PCN-80-773-s001.docx]

**Supplementary Materials**

**Table of Contents**

1. Supplementary Table S1. Generalized Estimation Equation (GEE) model results for SID hit-or-not to compare remitted TRS with non-remitted TRS patients

2. Supplementary Table S2. Generalized Estimation Equation (GEE) model results for anticipatory pleasure to compare remitted TRS with non-remitted TRS patients

3. Supplementary Table S3. Generalized Estimation Equation (GEE) model results for consummatory pleasure to compare remitted TRS with non-remitted TRS patients

4. Supplementary Table S4. Spearman’s correlations of change in mean scores of SID performance with the CAINS subscale scores in the clinical sample (n = 120)

5. Supplementary Table S5. Spearman’s correlations of change in mean SID anticipatory pleasure ratings with the CAINS subscale scores in TRS sample (n = 60)

6. Supplementary Table S6. Spearman’s correlations of change in mean SID anticipatory pleasure ratings with the CAINS subscale scores in remitted schizophrenia sample (n = 60)

**Supplementary Table S1. Generalized Estimation Equation (GEE) model results for SID hit-or-not to compare remitted TRS with non-remitted TRS patients**

| Model S1a (remitted TRS patients + non-remitted SCZ patients) | | | |
| --- | --- | --- | --- |
|  | beta-coefficient | SE | p-value |
| Subject | -1.33E-04 | 1.72E-04 | 0.44 |
| Negative cue | 2.83E-02 | 5.16E-02 | 0.583 |
| Positive Cue | -4.13E-02 | 5.08E-02 | 0.417 |
| Non-remitted TRS* | -5.92E-02 | 1.81E-01 | 0.743 |
| Sex (male) | -2.73E-01 | 1.27E-01 | **0.032** |
| Age | 3.34E-03 | 7.33E-03 | 0.649 |
| Trial number | 2.15E-02 | 3.46E-03 | **<0.001** |
| TONI nonverbal IQ | 5.26E-03 | 4.08E-03 | 0.198 |
| Anticholinergic burden | 4.88E-02 | 8.36E-02 | 0.559 |
| DDD (olanzapine, mg/d) | 3.31E-03 | 6.59E-03 | 0.616 |
| PANSS-positive | 3.77E-03 | 1.60E-02 | 0.813 |
| PANSS-general | -2.38E-02 | 2.39E-02 | 0.318 |
| CAINS-MAP | 1.57E-02 | 1.27E-02 | 0.217 |
| CAINS-EXP | -5.44E-02 | 1.88E-02 | **0.004** |
|  |  |  |  |
| Model S1b (remitted TRS patients + non-remitted SCZ patients) | | | |
|  | beta-coefficient | SE | p-value |
| Subject | -1.32E-04 | 1.72E-04 | 0.444 |
| Negative cue | 3.84E-02 | 8.94E-02 | 0.667 |
| Positive Cue | -1.43E-01 | 8.68E-02 | 0.099 |
| TRS | -1.08E-01 | 1.96E-01 | 0.581 |
| Sex (male) | 2.73E-01 | 1.27E-01 | **0.032** |
| Age | 3.34E-03 | 7.34E-03 | 0.649 |
| Trial number | 2.15E-02 | 3.46E-03 | **<0.001** |
| TONI nonverbal IQ | 5.31E-03 | 4.08E-03 | 0.193 |
| Anticholinergic burden | 4.87E-02 | 8.36E-02 | 0.56 |
| DDD (olanzapine, mg/d) | 3.33E-03 | 6.58E-03 | 0.612 |
| PANSS-positive | 4.12E-03 | 1.60E-02 | 0.797 |
| PANSS-general | -2.42E-02 | 2.39E-02 | 0.312 |
| CAINS-MAP | 1.57E-02 | 1.27E-02 | 0.217 |
| CAINS-EXP | -5.43E-02 | 1.89E-02 | **0.004** |
| Non-remitted TRS x Negative cue | -1.67E-02 | 1.10E-01 | 0.879 |
| Non-remitted TRS x Positive Cue | 1.65E-01 | 1.07E-01 | 0.123 |
|  |  |  |  |
| Note: TRS = treatment-resistant schizophrenia; SCZ = schizophrenia; PANSS = the Positive and Negative Symptom Scales; CAINS = the Clinical Assessment Interview for Negative Symptoms; DDD = daily-defined dose  * Remitted-TRS as the reference group | | | |

**Supplementary Table S2. Generalized Estimation Equation (GEE) model results for anticipatory pleasure to compare remitted TRS with non-remitted TRS patients**

| Model S2a (remitted TRS patients + non-remitted SCZ patients) | | | |
| --- | --- | --- | --- |
|  | beta-coefficient | SE | p-value |
| Subject | 2.16E-04 | 8.48E-05 | **0.011** |
| Negative cue | -1.53E-02 | 7.17E-03 | **0.032** |
| Positive Cue | -8.69E-03 | 5.78E-03 | 0.133 |
| Non-remitted TRS* | -2.44E-02 | 9.59E-02 | 0.799 |
| Sex (male) | 4.79E-02 | 7.26E-02 | 0.509 |
| Age | -2.11E-03 | 4.33E-03 | 0.625 |
| Trial number | 9.92E-04 | 5.10E-04 | 0.052 |
| TONI nonverbal IQ | -2.56E-03 | 2.57E-03 | 0.319 |
| Anticholinergic burden | -4.92E-02 | 2.48E-02 | **0.047** |
| DDD (olanzapine, mg/d) | 2.38E-03 | 3.04E-03 | 0.433 |
| PANSS-positive | 1.57E-02 | 6.82E-03 | **0.021** |
| PANSS-general | 3.67E-04 | 9.78E-03 | 0.970 |
| CAINS-MAP | -4.65E-03 | 5.62E-03 | 0.408 |
| CAINS-EXP | -1.14E-02 | 1.22E-02 | 0.349 |
|  |  |  |  |
| Model S2b (remitted TRS patients + non-remitted SCZ patients) | | | |
|  | beta-coefficient | SE | p-value |
| Subject | 2.15E-04 | 8.46E-05 | **0.011** |
| Negative cue | -2.96E-02 | 1.44E-02 | **0.040** |
| Positive Cue | -1.57E-02 | 1.28E-02 | 0.219 |
| TRS | -3.36E-02 | 9.57E-02 | 0.726 |
| Sex (male) | 4.71E-02 | 7.22E-02 | 0.514 |
| Age | -2.21E-03 | 4.29E-03 | 0.607 |
| Trial number | 9.92E-04 | 5.10E-04 | 0.052 |
| TONI nonverbal IQ | -2.50E-03 | 2.55E-03 | 0.326 |
| Anticholinergic burden | -4.87E-02 | 2.48E-02 | **0.049** |
| DDD (olanzapine, mg/d) | 2.45E-03 | 3.03E-03 | 0.419 |
| PANSS-positive | 1.57E-02 | 6.80E-03 | **0.021** |
| PANSS-general | 1.85E-04 | 9.76E-03 | 0.41 |
| CAINS-MAP | -4.64E-03 | 5.63E-03 | 0.41 |
| CAINS-EXP | -1.14E-02 | 1.22E-02 | 0.349 |
| Non-remitted TRS x Negative cue | 2.33E-02 | 1.68E-02 | 0.165 |
| Non-remitted TRS x Positive Cue | 1.15E-02 | 1.35E-02 | 0.397 |
|  |  |  |  |
| Note: TRS = treatment-resistant schizophrenia; SCZ = schizophrenia; PANSS = the Positive and Negative Symptom Scales; CAINS = the Clinical Assessment Interview for Negative Symptoms; DDD = daily-defined dose  * Remitted-TRS as the reference group | | | |

**Supplementary Table S3. Generalized Estimation Equation (GEE) model results for consummatory pleasure to compare remitted TRS with non-remitted TRS patients**

| Model S3a (remitted TRS patients + non-remitted SCZ patients) | | | |
| --- | --- | --- | --- |
|  | beta-coefficient | SE | p-value |
| Subject | 1.97E-04 | 8.13E-05 | **0.015** |
| Negative cue | -2.10E-02 | 7.67E-03 | **0.006** |
| Positive Cue | 2.97E-02 | 1.31E-02 | **0.024** |
| Non-remitted TRS* | 6.46E-02 | 8.91E-02 | 0.468 |
| Sex (male) | 6.91E-02 | 5.70E-02 | 0.225 |
| Age | -4.01E-03 | 3.20E-03 | 0.210 |
| Trial number | 2.14E-03 | 6.02E-04 | **<0.001** |
| TONI nonverbal IQ | -2.75E-04 | 2.53E-03 | 0.913 |
| Anticholinergic burden | 6.84E-03 | 2.69E-02 | 0.799 |
| DDD (olanzapine, mg/d) | -7.99E-04 | 2.72E-03 | 0.769 |
| PANSS-positive | 2.34E-02 | 7.37E-03 | **0.001** |
| PANSS-general | -3.08E-03 | 1.08E+00 | 0.774 |
| CAINS-MAP | -3.67E-03 | 5.53E-03 | 0.507 |
| CAINS-EXP | -1.20E-02 | 1.24E-02 | 0.337 |
|  |  |  |  |
| Model S3b (remitted TRS patients + non-remitted SCZ patients) | | | |
|  | beta-coefficient | SE | p-value |
| Subject | 1.98E-04 | 8.13E-05 | **0.015** |
| Negative cue | -3.66E-02 | 1.42E-02 | **0.010** |
| Positive Cue | 1.38E-02 | 2.86E-02 | 0.630 |
| TRS | 4.74E-02 | 9.20E-02 | 0.607 |
| Sex (male) | 6.94E-02 | 5.72E-02 | 0.225 |
| Age | -4.12E-03 | 3.19E-03 | 0.197 |
| Trial number | 2.14E-03 | 6.02E-04 | **<0.001** |
| TONI nonverbal IQ | -2.89E-04 | 2.52E-03 | 0.909 |
| Anticholinergic burden | 7.22E-03 | 2.68E-02 | 0.787 |
| DDD (olanzapine, mg/d) | -7.18E-04 | 2.72E-03 | 0.792 |
| PANSS-positive | 2.36E-02 | 7.33E-03 | **0.001** |
| PANSS-general | -3.30E-03 | 1.07E-02 | 0.759 |
| CAINS-MAP | -3.69E-03 | 5.54E-03 | 0.505 |
| CAINS-EXP | -1.20E-02 | 1.24E-02 | 0.335 |
| Non-remitted TRS x Negative cue | 2.49E-02 | 1.71E-02 | 0.146 |
| Non-remitted TRS x Positive Cue | 2.53E-02 | 3.10E-02 | 0.414 |
|  |  |  |  |
| Note: TRS = treatment-resistant schizophrenia; SCZ = schizophrenia; PANSS = the Positive and Negative Symptom Scales; CAINS = the Clinical Assessment Interview for Negative Symptoms; DDD = daily-defined dose  * Remitted-TRS as the reference group | | | |

**Supplementary Table S4. Spearman’s correlations of change in mean scores of SID performance with the CAINS subscale scores in the clinical sample (n = 120)**

|  |  | Change of SID accuracy (mean) | | Change of SID anticipatory pleasure (mean) | | Change of SID consummatory pleasure (mean) | |
| --- | --- | --- | --- | --- | --- | --- | --- |
|  |  | Positive cue minus Neutral cue | Negative cue minus Neutral cue | Positive cue minus Neutral cue | Negative cue minus Neutral cue | Positive cue minus Neutral cue | Negative cue minus Neutral cue |
| CAINS-MAP | r | -0.075 | -.213^*^ | -0.055 | -0.151 | -0.112 | 0.009 |
|  | p | 0.417 | **0.020** | 0.552 | 0.099 | 0.221 | 0.926 |
| CAINS-EXP | r | -0.135 | -.197^*^ | 0.014 | -.241^*^ | -0.172 | 0.013 |
|  | p | 0.142 | **0.031** | 0.877 | **0.008** | 0.061 | 0.887 |
| Note: CAINS = the Clinical Assessment Interview for Negative Symptoms; SID = Social incentive delay task  *The results should be interpreted with the limitations of multiple testing, and potential confounding effects of covariates | | | | | | | |

**Supplementary Table S5. Spearman’s correlations of change in mean SID anticipatory pleasure ratings with the CAINS subscale scores in TRS sample (n = 60)**

|  |  | Change of SID anticipatory pleasure (mean) | | Change of SID consummatory pleasure (mean) | |
| --- | --- | --- | --- | --- | --- |
|  |  | Positive cue minus Neutral cue | Negative cue minus Neutral cue | Positive cue minus Neutral cue | Negative cue minus Neutral cue |
| CAINS-MAP | *r* | 0.020 | -0.045 | 0.055 | -0.042 |
|  | *p* | 0.882 | 0.731 | 0.677 | 0.753 |
| CAINS-EXP | *r* | 0.093 | 0.033 | 0.007 | 0.088 |
|  | *p* | 0.480 | 0.805 | 0.961 | 0.505 |
| Note: CAINS = the Clinical Assessment Interview for Negative Symptoms; SID = Social incentive delay task | | | | | |

**Supplementary Table S6. Spearman’s correlations of change in mean SID anticipatory pleasure ratings with the CAINS subscale scores in remitted schizophrenia sample (n = 60)**

|  |  | Change of SID anticipatory pleasure (mean) | | Change of SID consummatory pleasure (mean) | |
| --- | --- | --- | --- | --- | --- |
|  |  | Positive cue minus Neutral cue | Negative cue minus Neutral cue | Positive cue minus Neutral cue | Negative cue minus Neutral cue |
| CAINS-MAP | *r* | -0.082 | -0.068 | -0.132 | 0.217 |
|  | *p* | 0.533 | 0.604 | 0.313 | 0.095 |
| CAINS-EXP | *r* | 0.037 | -.345^*^ | -0.094 | -0.001 |
|  | *p* | 0.779 | **0.007** | 0.476 | 0.992 |
| Note: CAINS = the Clinical Assessment Interview for Negative Symptoms; SID = Social incentive delay task  *The results should be interpreted with the limitations of multiple testing, and potential confounding effects of covariates | | | | | |
